# Adults with high social anhedonia have altered neural connectivity with ventral lateral prefrontal cortex when processing positive social signals

**DOI:** 10.3389/fnhum.2015.00469

**Published:** 2015-08-26

**Authors:** Hong Yin, Laura M. Tully, Sarah Hope Lincoln, Christine I. Hooker

**Affiliations:** ^1^Department of Psychology, Harvard UniversityCambridge, MA, USA; ^2^Psychiatry and Behavioral Sciences, University of California at DavisSacramento, CA, USA; ^3^Imaging Research Center, University of California at DavisSacramento, CA, USA

**Keywords:** social anhedonia, schizophrenia, cognitive control of emotion, ventral lateral prefrontal cortex, psychophysiological interaction, positive social emotion

## Abstract

Social anhedonia (SA) is a debilitating characteristic of schizophrenia, a common feature in individuals at psychosis-risk, and a vulnerability for developing schizophrenia-spectrum disorders. Prior work (Hooker et al., 2014) revealed neural deficits in the ventral lateral prefrontal cortex (VLPFC) when processing positive social cues in a community sample of people with high SA. Lower VLPFC neural activity was related to more severe self-reported schizophrenia-spectrum symptoms as well as the exacerbation of symptoms after social stress. In the current study, psycho-physiological interaction (PPI) analysis was applied to further investigate the neural mechanisms mediated by the VLPFC during emotion processing. PPI analysis revealed that, compared to low SA controls, participants with high SA exhibited reduced connectivity between the VLPFC and the motor cortex, the inferior parietal and the posterior temporal regions when viewing socially positive (relative to neutral) emotions. Across all participants, VLPFC connectivity correlated with behavioral and self-reported measures of attentional control, emotion management, and reward processing. Our results suggest that impairments to the VLPFC mediated neural circuitry underlie the cognitive and emotional deficits associated with social anhedonia, and may serve as neural targets for prevention and treatment of schizophrenia-spectrum disorders.

## Introduction

Social anhedonia (SA), the reduced pleasure from social interaction, is a debilitating characteristic of schizophrenia (Kring and Elis, 2013). High SA is associated with poor social functioning in people with schizophrenia as well as at risk for psychosis (Blanchard et al., 1998, 2011). In addition, SA is elevated in non-psychotic individuals who have a first-degree schizophrenia relative (Laurent et al., 2000). Healthy (non-psychiatric) adults with high SA have elevated levels of schizophrenia-spectrum characteristics (Blanchard et al., 2011), and young adults with high SA are more likely to develop schizophrenia-spectrum disorders later (Kwapil, 1998). Collectively, the data suggest that SA is not only an intermediate phenotype of schizophrenia but also a behavioral characteristic that may contribute to the risk, severity, and functional outcome of schizophrenia-spectrum disorders. However, because SA can develop from multiple sources, including social rejection (Baumeister and Leary, 1995) and secondary (i.e., peripheral) consequences of schizophrenia, such as depressed mood (Blanchard et al., 2001), anti-psychotic medication (Juckel et al., 2006) and internalized stigma (Yanos et al., 2008), identifying the relationship between SA and schizophrenia pathology is difficult. Elucidating the neural mechanisms of SA from non-psychiatric and medication naïve adults could provide valuable information about the neurodevelopment and pathophysiology of schizophrenia and facilitate the development of interventions that target the neural deficits underlying the symptoms.

Social interaction is a complex behavior that involves the coordination of multiple processes supported by different neural systems, including: (1) *social cue perception*, which relies on the fusiform gyrus, the lateral occipital cortex, and the temporal cortices; (2) *emotion processing*, which relies on the amygdala, the superior temporal sulcus (STS), the ventromedial potion of the prefrontal cortex (PFC), and the somatosensory-related cortices (SRC; Calder and Young, 2005; Ochsner et al., 2012); (3) *reward processing*, which relies on the orbital PFC, the ventral striatum, the anterior cingulate cortex and the ventromedial PFC (Haber and Knutson, 2010); and (4) *cognitive control*, which is recruited for emotion regulation, relies on the dorsal and ventral lateral prefrontal cortices (DLPFC, VLPFC), the dorsal anterior cingulate cortex, the medial PFC, and the superior and inferior parietal cortices (SPC, IPC; Ochsner et al., 2012; Tully and Niendam, 2014; Hooker, 2015). Interacting with others and building interpersonal relationships requires accurate perception of social and emotional cues, appropriate emotional responses, and coordination of emotion, reward, and cognitive control systems to generate and sustain representations of the positive experience which will guide future social engagement. SA could result from dysfunction in any one of these neural systems or the interactions among them.

Recent research suggests that SA in schizophrenia-spectrum disorders is, primarily, related to deficits in cognitive control mechanisms that help create, sustain and manipulate representations of positive social experiences (Strauss and Gold, 2012). It is well established that deficits in cognitive control skills, such as attentional control and working-memory, are a central feature of schizophrenia-spectrum disorders (Barch, 2005). Schizophrenia individuals with worse cognitive control skills report higher trait negative affect (Strauss et al., 2013). Healthy adults with high SA have decreased cognitive control of emotional information (Tully et al., 2012), and attentional control mediates the relationship between SA and social impairments (Tully et al., 2014). Further conceptualizations of SA propose that deficits in these cognitive control mechanisms result in difficulty creating, sustaining, and/or retrieving representations of past pleasurable experiences to motivate goal-directed behavior (Pizzagalli, 2010). A large body of work in the literature supports that, while “in-the-moment” responses to rewards (i.e., consummatory pleasure) may be relatively intact (Horan et al., 2006; Mote et al., 2014a,b), people with schizophrenia or at high risk (Schlosser et al., 2014) have difficulty anticipating positive events, and the reduced anticipatory pleasure is related to SA and other negative symptoms (Gard et al., 2007; Hooker et al., 2014). On the other hand, reduced consummatory pleasure in schizophrenia (Strauss et al., 2011) and decreased positive affect to emotional stimuli among schizotypy individuals (Cohen et al., 2012) have also been reported, suggesting compromised momentary experiencing in the schizophrenia spectrum. Despite the mixed results, overall evidence supports the proposal that impaired cognitive control of emotion impacts generating and maintaining internal representations of rewards (Gold et al., 2008) and contributes to SA related emotional and social dysfunctions.

Neuroimaging reveals that cognitive control of emotion involves the interaction between cognitive control and the emotion processing neural networks (Petersen and Posner, 2012; Tully and Niendam, 2014). Within the cognitive control network, the lateral prefrontal cortex (LPFC) functions as a major hub integrating distributed neural regions involved in multiple processes, including emotion experiencing and the control of emotional information on goal-directed behavior (Gyurak et al., 2011; Ochsner et al., 2012). Higher LPFC neural activity during laboratory-based emotional tasks (e.g., viewing the partner’s positive expressions) predicts better emotion regulation after interpersonal conflicts, indicating that LPFC activation influences real-life functioning. When performing reappraisal of aversive images, healthy adults with greater VLPFC activity reported better success, and the influence of VLPFC activity on reappraisal success was negatively mediated by amygdala activity and positively mediated by ventral striatal activity (Wager et al., 2008), suggesting that the VLPFC regulates through inhibitory and excitatory control of regions associated with emotion generation (Ochsner et al., 2012). Together these data suggest that VLPFC control-related mechanisms facilitate adaptive emotional response to social interactions through the up-regulation of positive affect and/or down-regulation of negative affect.

A recent study by Hooker et al. (2014) suggests that SA is associated with deficits in VLPFC control of positive affect. When viewing videos of interpersonally relevant emotional facial expressions, community-based adults with high SA displayed reduced VLPFC activity in response to positive social (versus neutral) expressions. Lower VLPFC neural activity was correlated with lower anticipatory pleasure. Furthermore, daily diaries over the following three weeks revealed that the interaction of high SA and low VLPFC neural activity to positive social expressions predicted worse paranoia, cognition, positive affect, and motivation/productivity, establishing connections between anhedonia, VLPFC function, and goal-directed behavior (daily productivity). One interpretation is that, during positive social encounters, the VLPFC modulates other neural regions to amplify (i.e., up-regulate) salient elements of the interaction (such as positive social cues or affective responses), which helps create and maintain internal representations of the experience that guide future social interactions. This interpretation proposes that SA is associated with deficits in the interaction between the VLPFC and other distributed neural regions when processing positive social signals.

The aim of the current study is to investigate this proposed neural mechanism by examining the neural connectivity between the VLPFC and other emotion processing regions in high SA adults during viewing positive social emotions (e.g., accepting, loving, caring, admiring, etc). Dysfunctional prefrontal-amygdala connectivity during facial emotion recognition, including both positive and negative expressions, has been observed in people with SA (Fakra et al., 2008) and at clinical high risk for psychosis (Gee et al., 2012; Lord et al., 2012). Altered connectivity between social and emotional processing regions at rest has been reported in first-degree relatives of schizophrenia patients, with lower connectivity related to worse social cognition and social functioning (Dodell-Feder et al., 2014a). These results support that aberrant neural connectivity contributes to social–emotional disturbances in psychotic and psychosis-prone populations, yet to our knowledge, there has been no report on the direct relationships between SA, VLPFC neural connectivity, and positive social emotions.

Here, we applied psycho-physiological interaction (PPI) analysis to the fMRI data from Hooker et al. (2014). PPI analysis examines changes in the strength of neural interactions as a function of the psychological task (Friston et al., 1997; Gitelman et al., 2003). It does not address causality but provides information on task-dependent coupling between neural regions. In this study, we defined the left VLPFC (identified in Hooker et al., 2014) as the seed region and used PPI analysis to identify brain areas that have stronger interaction with the VLPFC when viewing socially positive relative to neutral expressions. Positive PPI activity indicates elevated VLPFC connectivity for positive social emotions and is consistent with the hypothesis that the VLPFC is up-regulating activity in other regions. To further examine the functional impact of VLPFC connectivity, we conducted correlation analysis between VLPFC connectivity and behavioral measures related to cognitive control of emotion.

In summary, we performed PPI analysis to test the hypotheses that: (1) the VLPFC has greater connectivity with social, emotional, and reward processing regions when viewing socially positive relative to neutral expressions; (2) people with high SA have disrupted VLPFC connectivity for socially positive relative to neutral expressions; and (3) lower VLPFC connectivity with social, emotional, and reward processing regions is related to worse impairments in behavioral processes that are associated with these neural regions and are known to be deficient in high SA individuals.

## Materials and Methods

### Participants and Assessments

Details of participant recruitment and assessment are reported in Hooker et al. (2014). Briefly, thirty healthy adults who were primary English speakers and between ages 18–60 were recruited from the Greater Boston community. Participants were screened with the Structured Clinical Interview for DSM IV Axis I Disorders (SCID; First et al., 2002) and the Structured Clinical Interview for DSM IV Personality Disorders (First et al., 1997)^1^. Participants were evaluated on the Revised Social Anhedonia Scale (RSAS; Eckblad et al., unpublished test) and categorized as high SA if their RSAS score was over 1.96SD above the population mean; or low SA if RSAS score was equal to or less than 1 SD above the population mean. Exclusion criteria included: IQ < 70, head trauma^2^, neurological illness, substance abuse within 6 months, or current/past Axis I or II disorder^3^. All participants gave written informed consent in accordance to the guidelines provided by the IRB at Harvard University.

To assess social, emotion and reward processing, participants completed laboratory assessments and self-reported questionnaires including the Temporal Experience of Pleasure Scale (TEPS; Gard et al., 2006), the Managing Emotions (ME) subscale of the Mayer–Salovey–Caruso Emotional Intelligence Test (MSCEIT-ME; Mayer et al., 2003), the Attentional Network Task-Emotion version (ANT-E; Tully et al., 2012), and the Global Functioning: Social scale (GF: Social; Cornblatt et al., 2007).

The TEPS is a self-report questionnaire that includes consummatory and anticipatory subscales measuring trait tendencies in experiencing and anticipating pleasure (Gard et al., 2006); statements such as “The smell of freshly cut grass is enjoyable to me” (consummatory) and “When something exciting is coming up in my life, I really look forward to it” (anticipatory) are rated on a 1–6 Likert scale with higher scores indicating more hedonic responses. The MSCEIT (Mayer et al., 2002) is a standardized, performance-based test of emotion processing. The ME subscale includes two types of tasks that measure the ability to understand and manage emotions and social situations. Participants read a short vignette or statement and determine the best response from several presented options – e.g., “Mara woke up feeling pretty well. How well would each action help her preserve her mood?” and “John’s close friend at work had taken a new job and would be moving out of the area. How effective would John be in maintaining a good relationship if he chose to respond in each of the following ways?” The ANT-E task is a performance-based flanker task measuring the cognitive control of emotion. Participants determine the direction of an arrow flanked by either angry or neutral faces by focusing on the arrow (the target) while inhibiting the distraction from the nearby faces. Cognitive control of emotion was measured by calculating the difference in reaction time between the tasks with angry and neutral face flankers as (reaction time with angry faces – reaction time with neutral faces), with higher values indicating poorer inhibition control over the distraction of angry faces. The GF: Social scale is an interviewer-rated assessment of social functioning; participants receive a score of 1-to-10 with 10 indicating superior social functioning and 1 indicating extreme social dysfunction.

### fMRI Tasks and Experimental Design

The fMRI experiment used a blocked design. Participants viewed blocks of videos depicting one of the three types of interpersonally relevant facial expressions: socially positive (accepting), neutral, or socially negative (rejecting). Each block was 24 s long and contained six short videos (each 3 s long) of the same emotion type with different facial identities. Before the scan, participants were told to “imagine you are interacting with this person.” At the end of each block, participants were asked to answer the question “How accepted or rejected do you feel?” on a 5-point scale (1 = Very rejected; 3 = Neutral; 5 = Very accepted). The response scale was presented for 3 s followed by 12 s rest (See Hooker et al., 2014). An fMRI scan session included three runs each containing four blocks of each emotion type with semi-random block sequences.

### Neuroimaging

Magnetic resonance imaging scans were conducted on a Siemens 3T Tim Trio scanner with a whole head, 12-channel head coil at the Center for Brain Science at Harvard University, Cambridge, MA, USA. For each participant, a series of T2^∗^ weighted functional MRI volumes were obtained. Each fMRI volume was acquired by using 40 interleaved oblique-axial slices with voxel size of 3 mm × 3 mm × 3 mm, TE of 30 ms, flip angle of 85°, field of view (FOV) of 216 mm × 216 mm and acquisition repetition time (TR) of 2560 ms. Anatomical T1 weighted high resolution (MEMPRAGE) scan was obtained with 176 axial slices with voxel size of 1 mm × 1 mm × 1 mm, TE of 7.22 ms, flip angle of 7°, FOV of 256 mm × 256 mm and TR of 2530 ms.

### Image Processing and Data Analysis

Preprocessing of imaging data and subject level statistical analysis using general linear model (GLM) were carried out in SPM8 (Wellcome Trust Centre for Neuroimaging, University College of London, UK); details as described in Hooker et al. (2014). In summary, scan data were corrected for slice-timing, realigned to mean image via a two-pass procedure and co-registered to the T1 high resolution image. During realignment, head motion relative to the first volume in each run was estimated. T1 high resolution structural scan was subsequently segmented and used to normalize the functional scans to standard Montreal Neurological Institute (MNI) space. The normalized images were then smoothed with an 8 mm full width at half maximum (FWHM) Gaussian kernel and high-pass filtered with cutoff of 0.008 (1/128 s) Hz. Artifact outliers (defined as those with volume-to-volume combined head motion >3 mm (*x*, *y*, and *z*) or 0.02° (pitch, roll, and yaw), or image intensity change >4 SD from the global mean of each run) were identified by Artifact Detection Tool^4^. Task conditions were modeled by using a boxcar function convolved with the canonical hemodynamic response function provided by SPM8. Effects of psychological tasks at the single subject level were estimated by GLM, with head motion and artifact outliers as covariates of no interest. Statistical T maps and F contrast map of all effects (including all tasks and covariates) were computed for each participant.

Whole brain (seed to voxel) PPI analysis was conducted according to the manual provided by SPM8 (Wellcome Trust Centre for Neuroimaging, University College of London, UK^5^ (Gitelman et al., 2003). Based on the prior data (Hooker et al., 2014), the seed region of interest (ROI) was determined as a sphere of 8 mm radius centered at the left VLPFC (MNI: [–33, 41, 13]) – i.e., the region in which high SA participants had reduced activity for socially positive versus neutral expressions. We first displayed the all-effect F map (*p* < 0.05, FWE corrected) and then extracted the eigenvariate time course of neural activity in the left VLPFC region for each subject, adjusting for the all-effect F contrast. PPI toolbox in SPM8 was then used to construct a time course vector (PPI vector) of the interaction between neural activity in the seed region and each of the following psychological task conditions: viewing socially positive, neutral, as well as viewing socially positive relative to neutral emotions (i.e., Positive > baseline, Neutral > baseline and Positive > Neutral, respectively).

To identify neural regions temporally correlated with each PPI time course at the individual subject level, each PPI vector, together with the main effects of neural activity and the related psychological task condition, were entered as regressors into a GLM. Head motion and artifact outliers were included as covariates of no interest. Contrast maps of voxels correlating to the seed PPI time course under Positive > Neutral, Positive > baseline or Neutral > baseline conditions were generated for each participant.

To assess group level effects under the Positive > Neutral condition, participant’s (Positive > Neutral) PPI contrast maps were entered into one-sample T (for within-group analysis, **Table 2**) and independent two-sample *t*-tests (for between group analysis, **Table 3**). To compare group level differences under the conditions of Positive > baseline or Neutral > baseline, each participant’s contrast maps of those conditions were entered into a 2 group (low and high SA) × 2 condition (Positive > baseline and Neutral > baseline) ANOVA and the results were presented in Supplementary Figure S2.

Unless specified otherwise, imaging results were thresholded at *p* < 0.001 uncorrected for multiple comparison with cluster extent set at the value larger than the expected voxels per cluster (*k*e ≥ 8 voxels/216 mm^3^), which is computed in SPM8 based on random field analysis and reflects the average cluster size due to random noise (Friston et al., 1994). All supra-threshold clusters in the between-group comparison under the Positive > Neutral condition (**Table 3**) were subsequently corrected for multiple comparison by small volume correction (SVC) using masks of standard gray matter regions defined by WFUPickatlas toolbox in SPM. When a peak cluster fell on the border between two defined anatomical regions, such as the posterior region of the ITS or STS, masks were manually created that included the sulcus and neighboring gyri regions (see Supplementary Figure S1). Cluster size was reported in mm^3^.

### Correlation Analysis

The mean PPI activity value (i.e., beta value) of each cluster identified in the between group analysis (listed in **Table 3**) was extracted using the REX toolkit^6^. These values were then used in correlation analysis to examine the relationship between VLPFC neural interactions and measures of social cognition and functioning. Using SPSS (version 22), partial correlations, controlling for SA score, were conducted between the assessed measures and PPI activity. Controlling for the participant’s SA score was necessary to remove the confounding effect of sample selection. Results are reported in **Table 4**. Bonferroni correction for multiple tests was conducted and correlations that remained significant (*p* < 0.05) after Bonferroni correction are denoted with ^b^ (**Table 4**).

## Results

### Participant Characteristics

Characteristics of the participants are listed in **Table 1** (also see Hooker et al., 2014). Low SA and high SA participants were matched in age and gender. Significant differences were observed between the two groups in the TEPS anticipatory and GF Social scale. No group difference was observed on the ANT-E, TEPS consummatory or MSCEIT-ME. fMRI task ratings indicated that participants felt “accepted” when viewing positive social emotions [ratings mean (SD), low SA: 4.71 (0.42); high SA: 4.46 (0.44); between group test: *t*(29) = 1.5, *p* = 0.14] and felt neural when viewing neutral expressions [ratings mean (SD), low SA: 3.05 (0.39); high SA: 2.89 (0.28); between group test: *t*(29) = 1.3, *p* = 0.21], with no significant between group differences (Hooker et al., 2014).

**Table 1:** Participant characteristics.

Behavioral assessment	Low Social Anhedonia (SA)	High SA	Two-Sample *t*-test
		
	Mean (SD) [range]	Mean (SD) [range]	*t*-value (df)*p*-value
Gender (F/M)^†^	10/5	8/7	χ^2^ = 0.56, (*p* = 0.46)
Age^†^	30.27 (10.47) [19–51]	32.00 (12.75) [20–52]	*t* = -0.407 (28)*p* = 0.687
Social Anhedonia	2.67 (2.53) [0–10 ]	24.60 (5.63) [17–38 ]	*t* = -13.768 (19.42) ^¶^ *p* < 0.001^∗∗∗^
Temporal Experience of Pleasure Scale (TEPS)-Anticipatory^§†^	46.00 (4.90) [37–54]	36.86 (5.68) [29–44]	*t* = 4.650 (27) *p* < 0.001^∗∗∗^
TEPS-Consummatory^§†^	36.27 (5.47) [20–41]	32.86 (7.75) [15–42]	*t* = 1.376 (27) *p* = 0.180
Attentional Network Task-Emotion (ANT-E) reaction time (ms)	2.71 (14.47) [-23.73–28.83]	12.34 (20.51) [-9.92–55.79]	*t* = -1.486 (28) *p* = 0.148
Global Social Functioning^§†^	9.40 (0.74) [8–10]	6.79 (1.63) [3–9]	*t* = 5.512 (17.85) ^¶^ *p* < 0.001^∗∗∗^
Mayer–Salovey–Caruso Emotional Intelligence Test (MSCEIT-ME)	99.81 (9.55) [77.82–111.64]	96.51 (8.11) [73.88–106.78]	*t* = 1.021 (28) *p* = 0.316

*Independent two-sample t-tests were used to compare group means, assuming equal variance of each group unless Levene’s variance equality test indicated significant difference in variance, in which case non-equal variance was assumed. ^§^ Missing data for one high SA participant; ^†^Data reported previously (Hooker et al., 2014); ^∗∗∗^*p* < 0.001 (two-tailed); ^¶^ Non-equal variance assumed in two-sample *t*-test*.

### Within-Group Analysis of PPI Activity When Viewing Positive Social Emotions

We first examined left VLPFC connectivity within each group separately. In the low SA group, PPI analyses for Positive > Neutral condition revealed positive neural interactions between the left VLPFC region and the left supramarginal cortex and between the left VLPFC and the left superior and middle frontal cortices. No supra-threshold clusters were observed for the reverse condition of Neutral > Positive (see **Table 2**, Supplementary Figure S3). Positive PPI activity between the VLPFC and regions of emotion processing and cognitive control was observed under both the Positive > baseline and Neutral > baseline conditions (Supplementary Figure S2). In short, the low SA group displayed elevated VLPFC connectivity when viewing positive social expressions relative to neutral expressions.

**Table 2:** Psycho-physiological interaction (PPI) activity for viewing positive social emotions (versus neutral emotion).

Region	L/R	BA	Montreal Neurological Institute (MNI) Coordinates	Cluster Size (mm^3^)	*t*-value
**Low SA, PPI of Positive > Neutral**					
Supramarginal Cortex	L	BA2	[-63, -31, 31]	513	5.50
Superior Frontal Cortex	L	BA8	[-21, 5, 49]	729	5.06
Superior Frontal Cortex	L	BA8	[-18, 17, 61]	243	4.62
Middle Frontal Cortex	L	BA46	[-33, 41, 25]	243	4.23
**Low SA, PPI of Positive < Neutral**					
No significant cluster					
**High SA, PPI of Positive > Neutral**					
No significant cluster					
**High SA, PPI of Positive < Neutral**					
Inferior Occipital Cortex	L	BA19	[-39, -70, -5]	5562	8.36
Inferior Occipital Cortex	R	BA19	[39, -73, -5]	8289	6.86
Lingual	R	BA17	[15, -88, -2]	1836	6.43
Middle Temporal Gyrus/Superior Temporal Sulcus (STS)	R	BA22	[51, -34, 4]	1620	5.46
Inferior Frontal Operculum	R	BA44	[45, 8, 22]	945	5.21
Inferior Frontal Operculum	L	BA44	[-48, 5, 28]	1917	5.02
Inferior Frontal Triangularis	R	BA48	[39, 26, 16]	513	4.78
Inferior Frontal Triangularis	L	BA48	[-36, 32, 7]	297	4.74
Amygdala	L	BA35	[-18, -10, -14]	297	4.37

*Seed region was a sphere of 8 mm radius centered at MNI coordinates [-33, 41, 13]. Shown are one-sample *t*-test results of the Positive > Neutral contrasts for low SA or high SA group (*p* < 0.001, uncorrected, *k* = 216 mm^3^), respectively. BA, Brodmann area; L/R, laterality*.

The high SA group showed a different PPI connectivity pattern from the same VLPFC seed. No supra-threshold PPI clusters were observed under the Positive > Neutral condition (**Table 2**, Supplementary Figure S3), Under the Neutral > Positive condition, significant bilateral clusters of PPI activity were observed in inferior occipital cortex including inferior temporal and fusiform cortices, inferior frontal operculum and inferior frontal triangularis. Supra-threshold clusters were also observed in the right lingual, right STS and left amygdala. In general, the high SA group displayed reduced VLPFC mediated connectivity when viewing socially positive (versus neutral) emotion.

### Between-Group Comparison of PPI Activity When Viewing Positive Social Emotions

**FIGURE 1:**
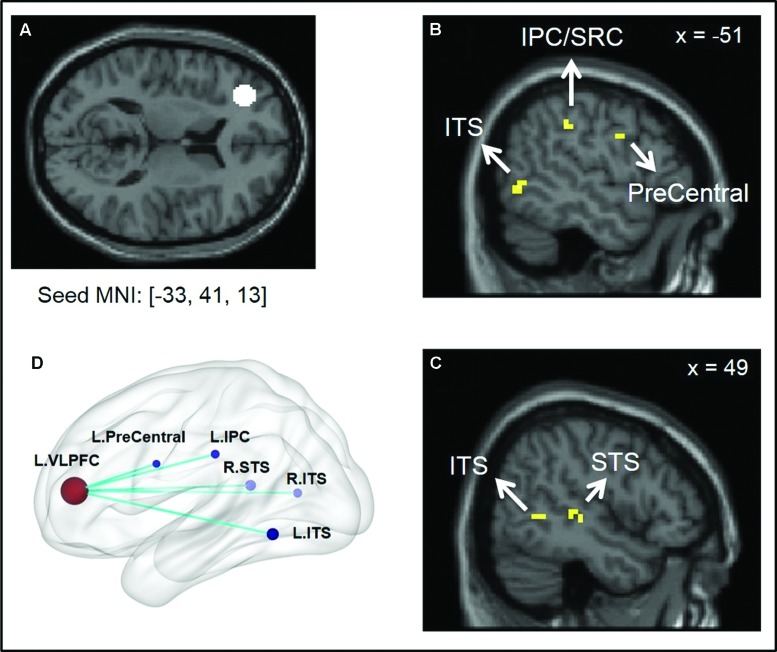
**Illustration of psycho-physiological interaction (PPI) results showing seed region and regions it interacted with. (A)** Ventral lateral prefrontal cortex (VLPFC) seed region of 8 mm radius sphere centered at Montreal Neurological Institute (MNI) coordinates [–33, 41, 13]; **(B,C)** Regions showing stronger interaction to VLPFC seed under low SA > high SA and Positive > Neutral conditions. **(D)** Surface rendering of PPI seed and interaction regions (left side closer to the viewer) visualized with BrainNet Viewer (http://www.nitrc.org/projects/bnv/), (Xia et al., 2013).

The between-group comparison of PPI activity under the Positive > Neutral condition showed that, compared to the low SA group, the high SA group exhibited reduced neural connectivity between the left VLPFC and the following regions: left inferior parietal cortex (IPC/SRC), left precentral gyrus/motor cortex, bilateral inferior temporal sulcus (ITS), and right STS (**Figure 1** and **Table 3**)^7^. No supra-threshold clusters were observed for the reverse condition (i.e., high SA > low SA for Positive > Neutral). Between-group comparisons of each condition separately showed no significant difference between low and high SA for Positive > baseline and Neutral > baseline conditions (Supplementary Table S1). These findings indicate that the between group PPI difference for Positive > Neutral reflects inverse connectivity patterns in the two groups; specifically, more VLPFC connectivity for positive social emotions versus neutral was observed in the low SA group, while the opposite (i.e., more VLPFC connectivity for neutral versus positive social expressions) was observed in high SA group.

**Table 3:** Comparison of PPI activity between high SA and low SA groups.

Region	L/R	BA	MNI Coordinates	Cluster Size (mm^3^)
**Low SA > high SA, Positive > Neutral**				
Inferior parietal cortex/Somatosensory cortex	L	BA40/BA2	[-48, -34, 37]	351
Precentral gyrus/Premotor cortex	L	BA6	[-48, -4, 31]	270
Middle temporal gyrus/Inferior temporal sulcus (ITS)	L	BA37	[-51, -64, -2]	378
STS	R	BA22	[51, -31, 4]	540
ITS/Fusiform cortex	R	BA37	[51, -55, 1]	378^†^
**Low SA < high SA, Positive > Neutral**				
No significant cluster				

*Shown are two-sample *t*-test results of PPI activity for viewing positive social emotions (versus neutral emotion). Data were thresholded at *p* < 0.001 (uncorrected, *k* = 216 mm^3^). PPI seed region was a sphere of 8 mm radius centered at MNI [-33, 41, 13]. Reported are gray matter clusters that survived cluster peak level small volume correction (*p* < 0.05, FWE corrected), with one cluster thresholded at *p* < 0.051 (FWE corrected), marked with ^†^. BA, Brodmann area; L/R, laterality*.

### Correlation Analysis

Results of the Spearman rank correlation analysis are listed in **Table 4**. Across all participants, Spearman partial correlation analysis controlling for SA score showed: (1) a positive correlation between left VLPFC ↔ right STS connectivity and the TEPS anticipatory pleasure; (2) a positive correlation between left VLPFC ↔ left IPC/SRC connectivity and the MECEIT-ME; and (3) negative correlations between the ANT-E reaction time and left VLPFC ↔ left IPC/SRC as well as left VLPFC ↔ left ITS connectivity, respectively. The correlation between the ANT-E and left VLPFC ↔ left IPC/SRC connectivity remained significant after Bonferroni multiple comparison correction. (4) No significant correlation was observed between VLPFC PPI activity and social functioning (GF: Social scale).

**Table 4:** Correlation of ventral lateral prefrontal cortex (VLPFC) PPI activity and behavioral measures.

PPI interaction	TEPS-A^§^	TEPS-C^§^	GF: Social^§^	MSCEIT-ME	ANT-E reaction time
VLPFC [-33, 41, 13] ↔ STS [51, -31, 4]	0.381^∗^	0.074	0.272	-0.059	-0.145
VLPFC [-33, 41, 13] ↔ ITS [-51, -64, -2]	-0.029	0.023	-0.002	0.290	-0.497^∗∗^
VLPFC [-33, 41, 13] ↔ IPC [-48, -34, 37]	0.319	0.033	0.055	0.409^∗^	-0.585^∗∗b^

*Shown are Spearman rank correlation coefficients from partial correlations after controlling for social anhedonia score. Only clusters with significant correlation to at least one measure are shown. PPI seed region was a sphere of 8 mm radius centered at MNI [-33, 41, 13]. PPI interaction regions were the clusters reported in **Table 3**. TEPS-A, TEPS anticipatory; TEPS-C, TEPS consummatory; GF: Social, global social functioning; MSCEIT-ME, MSCEIT branch of emotion and social management. ^∗^*p* < 0.05; ^∗∗^*p* < 0.01; ^b^significant after Bonferroni correction; ^§^ missing data for one high SA participant*.

## Discussion

The current study investigated whether SA is associated with disrupted connectivity between the VLPFC and other brain regions during the automatic processing of positive emotions in an interpersonal interaction. Results revealed three main findings: First, PPI analysis of the low SA group (i.e., participants with SA scores in the normal range) revealed stronger interactions between the left VLPFC and regions in the cognitive control and emotion processing networks when viewing positive social (versus neutral) expressions, consistent with the hypothesis that the VLPFC exerts cognitive control during emotion processing (Ochsner et al., 2012). Second, the degree of task-dependent modulation of VLPFC connectivity – i.e., the relative increase in VLPFC connectivity for socially positive relative to neutral expressions, was greater for low SA as compared to high SA participants. Third, correlation analyses showed that, across all participants, greater VLPFC connectivity for positive social (versus neutral) emotions was related to behavioral measures that rely on the cognitive control of emotion, including the self-reported tendency to anticipate reward (TEPS-Anticipatory), the ability to inhibit irrelevant emotional information (ANT-E), and the ability to understand and reason about emotions in individual and social contexts (MSCEIT-ME). Together the data supports our hypotheses that VLPFC mediated up-regulation of positive social emotions is compromised in people with high SA and this disrupted VLPFC connectivity underlies the behavioral impairments in social, emotion, and reward processing associated with high SA.

These findings add to the existing literature on the regulatory role of the VLPFC in emotion processing. Thus far, most research examines the down-regulation of negative emotion through explicit strategies, such as reappraisal, within an individual (non-social) context. Instead, our study elucidates VLPFC function during the automatic processing of positive emotions in a social context. PPI analysis in the low SA group provided information about normal VLPFC function. These results revealed neural interactions between the VLPFC and regions involved in emotion perception and cognitive control (Haxby et al., 2000; Calder and Young, 2005). Under the Positive > Neutral condition, increased connectivity was observed between the VLPFC and the supramarginal cortex within the general IPC/SRC region (**Table 2**). The IPC/SRC is considered part of the mirror neuron network (Caramazza et al., 2014) that promotes action embodiment and emotion perception. Lesions in the SRC are associated with impaired recognition of emotions, demonstrating that emotion perception requires intact representations generated in the SRC (Adolphs et al., 2000). The VLPFC also displayed positive coupling to the superior and middle frontal cortices, regions in the cognitive control network that facilitate attentional control and goal maintenance (D’Esposito, 2007; Buschman and Miller, 2014; Strauss et al., 2014). Taken together, PPI results from the low SA group demonstrated stronger VLPFC connectivity when viewing positive social (versus neutral) emotions, which we interpret as VLPFC up-regulation of neural regions involved in generating and sustaining representations of positive social cues.

Compared to the low SA group, under the Positive > Neutral condition, the high SA group showed reduced VLPFC connectivity to multiple regions (**Table 3**) including the posterior ITS (adjacent to fusiform face processing area; Ishai, 2008), and STS. Both the posterior ITS and STS are involved in face and emotion perception. The ITS/fusiform cortex is also part of the ventral stimulus-driven pathway of the attentional control network that detects visual stimuli and alerts frontal cognitive control regions (Corbetta and Shulman, 2002). The STS is considered the integration point of multiple modalities of sensory inputs, including physical and biological form and motion (Calder and Young, 2005). Reduced VLPFC modulation on those regions in high SA individuals may compromise the perception and encoding of positive social and emotional cues, and contribute to the emotion recognition deficits observed in schizophrenia-spectrum populations (Hooker and Park, 2002; Germine et al., 2011; Kring and Elis, 2013). Additionally, the high SA group displayed disrupted VLPFC connectivity with the precentral gyrus as well as the IPC/SRC. Both regions are part of the mirror neuron system responsible for generating physical and emotional embodiment of external stimuli. Thus, weak representations of social stimuli due to disrupted VLPFC regulation on mirror neuron regions could also contribute to deficits in social perception and emotion recognition. This is in line with our prior report showing that high SA individuals have abnormally low neural activity in the SRC, superior temporal gyrus and fusiform regions during facial emotion recognition (Germine et al., 2011). Additionally, disturbances in somatosensory processing are associated with psychosis and psychosis-proneness and may be a liability for developing schizophrenia (Chapman et al., 1978; Germine et al., 2013). Overall, reduced VLPFC connectivity when viewing positive social (versus neutral) expressions in the high SA group indicated weaker VLPFC regulation on regions responsible for perception and embodiment of positive social cues, which could lead to diminished internal representations of the stimuli that could, in turn, compromise the interpretation of social signals and impact the motivation for future interactions.

Results showing correlations between VLPFC connectivity and behavioral measures of social, emotion, and reward processing support our hypothesis that reduced VLPFC connectivity during social–emotional processing reflects a deficit in cognitive control of emotion which contributes to the social and emotional impairments associated with SA. Prior reports have established that schizophrenia-spectrum populations, including high SA individuals, have behavioral deficits in anticipatory reward, inhibitory control, and emotion management as assessed by a variety of instruments, including the TEPS-Anticipatory, ANT-E, and MSCEIT-ME scale (Dodell-Feder et al., 2014b; Hooker et al., 2014). Our data suggests that the strength of VLPFC connectivity underlies the subjective experience and/or behavioral performance in each of these functional domains. The negative correlation between VLPFC connectivity (VLPFC ↔ ITS/fusiform and VLPFC ↔ IPC/SRC, respectively) and the ANT-E reaction time indicates that stronger VLPFC connectivity is associated with faster ANT-E reaction time and, therefore, better inhibitory control over negative emotional distractions. This is consistent with the VLPFC’s regulatory role in selecting goal-relevant and suppressing irrelevant information and suggests that the VLPFC regulates activity in the SRC and ITS to either amplify internal representations of positive social cues (when viewing positive social emotions) or to suppress neural responses to emotional distractions (during the ANT-E tasks) depending on explicit or implicit regulatory goals.

The positive correlations of VLPFC connectivity to the TEPS-anticipatory and MSCEIT-ME scores further support the proposed VLPFC neural mechanisms. Anticipating pleasure requires successful retrieval of internal representations of past positive experiences. Emotion management, particularly as assessed by the MSCEIT-ME subscale, requires generating, maintaining, and manipulating emotions to predict how behavior and emotions influence each other in individual and social contexts. Thus both the TEPS-Anticipatory and MSCEIT-ME require or reflect the ability to retrieve and manipulate representations, which is a VLPFC function. The relationship between VLPFC connectivity and these two measures suggests that reduced VLPFC up-regulation of neural responses to positive social stimuli in high SA contributes to weakened representations of positive social interactions that subsequently compromise one’s ability to generate motivation for future reward and manage complex emotional and social situations.

Limitations of this study must be acknowledged. First, PPI analysis cannot address causality. Although we interpret our results as consistent with VLPFC regulation on emotion processing regions, we cannot exclude the possibility that the VLPFC was reversely regulated upon by the other regions. Further work using analysis method such as dynamic causal modeling (DCM) will help elucidate the causal relationships of the neural interactions. Secondly, the low sample size limits statistical power as well as generalizability of our results. Thirdly, aspects of the fMRI tasks could have contributed to some null results. Although several papers have reported aberrant connectivity in schizophrenia between the PFC and two core emotion generation regions – the amygdala and ventral striatum (Barch and Dowd, 2010; Anticevic et al., 2012), we found no marked group differences in VLPFC connectivity to those regions for the Positive > Neutral condition. Furthermore, there were no overall group differences in the Positive > baseline (one cluster in Neutral > baseline) condition. Viewing emotions without explicit instruction to regulate emotional responses might not have elicited strong enough VLPFC interactions to generate detectable group differences. In addition, different individual strategies employed during the tasks could have contributed to data variability that may have also limited detection power. Moreover, variations in neural responses to neutral expressions may obscure our interpretation of the observed PPI group difference at Positive > Neutral. Future research using larger sample sizes and a non-emotional task (e.g., viewing objects) as the control condition (i.e., Positive > Objects) may provide more specific information about SA related neural deficits during processing of positive social signals. Lastly, correlation analysis using single PPI connectivity values may not be sufficient to model relationships between neural connectivity and complex behaviors, such as social functioning, which could explain the lack of correlation between VLPFC connectivity and the GF: Social score. Multivariate approaches may offer higher sensitivity in identifying neural predictors for behavioral outcomes.

In summary, PPI analysis revealed neural mechanisms supporting the regulatory role of the VLPFC in social and emotional processing, especially in up-regulating responses to positive social signals. VLPFC mediated neural circuitry was disrupted in individuals with high SA and deficits in VLPFC connectivity were associated with impaired cognitive control of emotion. As SA is both a contributing factor to the risk for psychosis and a trait feature of schizophrenia, the VLPFC mediated neural mechanisms may represent potential targets for prevention and treatment of schizophrenia.

## Author Contributions

LT, SL, and CH designed research, LT and SL recruited participants and conducted the research; HY and CH performed fMRI analysis and wrote the paper; all authors contributed to the final manuscript.

## Conflict of Interest Statement

The authors declare that the research was conducted in the absence of any commercial or financial relationships that could be construed as a potential conflict of interest.

## Footnotes

1 Structured Clinical Interview for DSM IV Axis I Disorders (SCID) interviews were administered by advanced clinical psychology graduate students (SL, LT) and supervised by a licensed clinical psychologist (CH).

2 Head trauma was defined as a past major head injury involving a loss of consciousness lasting more than 20 min.

3 Head trauma and neurological illness were identified through health information questionnaires and then followed up by a medical history interview.

4 http://www.nitrc.org/projects/artifact_detect/

5 http://www.fil.ion.ucl.ac.uk/spm/

6 http://www.nitrc.org/projects/rex

7 One supra-threshold cluster was observed in white matter (MNI: [24, 2, 40], *k* = 270 mm^3^). As the interpretation of white matter fMRI is controversial in the literature (Gawryluk et al., 2014), it’s unclear whether this cluster reflected artifacts or true neural interactions. Thus it was excluded from **Table 3**.
