# Supplementary material for: Adults with high social anhedonia have altered neural connectivity with ventral lateral prefrontal cortex when processing positive social signals
Source: Front Hum Neurosci. 2015 Aug 26;9:469. doi: 10.3389/fnhum.2015.00469 (PMC4549656; doi:10.3389/fnhum.2015.00469)
Supplement: Supplementary file 1 [file Data_Sheet_2.PDF]

Supplementary Material

Adults with high social anhedonia have altered neural connectivity with ventral lateral prefrontal cortex when processing positive social signals

Hong Yin[1], Laura M. Tully[2], Sarah Hope Lincoln[1], Christine I. Hooker[1*]

[1] Department of Psychology, Harvard University, Cambridge, MA 02138, USA.
[2] Psychiatry and Behavioral Sciences, University of California, Davis, Sacramento, CA 95817, USA

*** Correspondence:** Christine I. Hooker, Department of Psychology, Harvard University, 1020 William James Hall, 33 Kirkland St, Cambridge, MA 02138, USA.
E-mail: chooker@wjh.harvard.edu

Supplementary Table

Table S1 Group comparison of PPI activity in a 2 group (low SA and high SA) x 2 condition (Positive>baseline and Neutral>baseline) ANOVA. Results were thresholded at $p<0.001$, uncorrected, k=216 mm^3. Seed region was a sphere of 8 mm radius centered at the left VLPFC (MNI: [-33, 41, 13]). BA: Brodmann area; L/R: laterality.

Region	L/R	BA	MNI Coordinates	Cluster Size (mm^3)	T-value
**low SA > high SA, Positive > baseline**					
No significant cluster					
**low SA < high SA, Positive > baseline**					
No significant cluster					
**low SA > high SA, Neutral > baseline**					
No significant cluster					
**low SA < high SA, Neutral > baseline**					
Inferior Frontal Triangularis	L	BA45	[-45, 35, 16]	351	3.92

Supplementary Figures

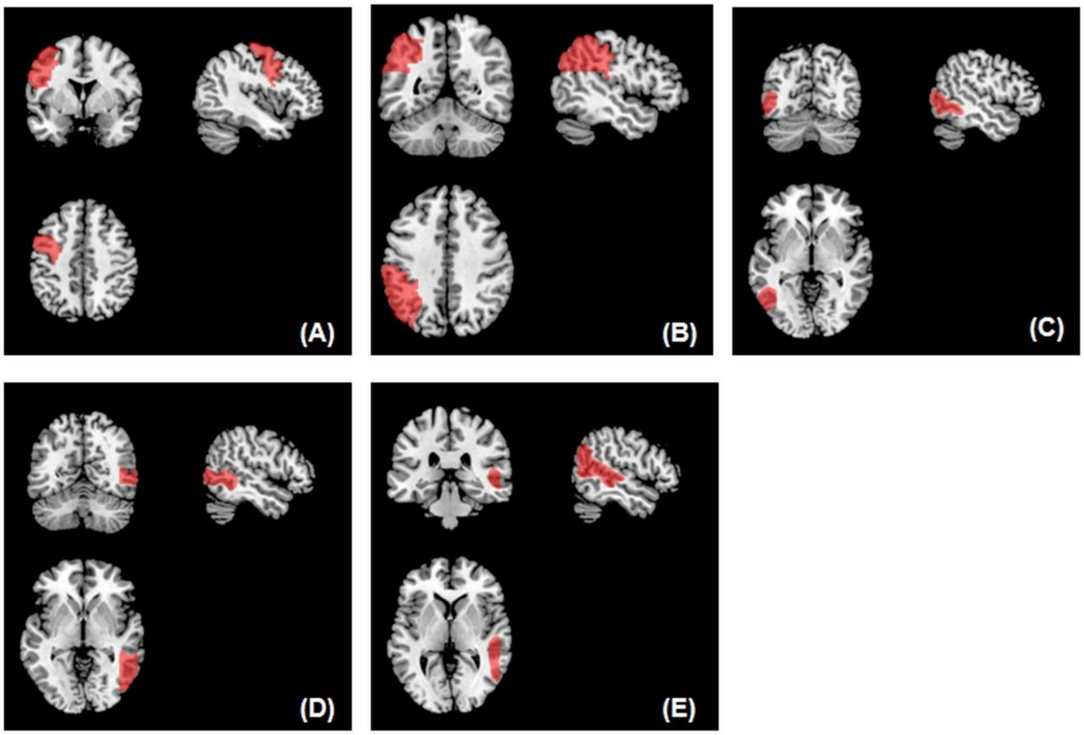

Figure S1 Masks used in small volume correction (SVC). A) Left precentral gyrus (WFU aal atlas); B) Left inferior parietal cortex-supramarginal cortex-angular gyrus (WFU aal atlas); C) left posterior middle temporal gyrus/inferior temporal sulcus; D) Right posterior inferior temporal sulcus; E) Right posterior superior temporal sulcus. All clusters reported in Table 3 also survived SVC with a sphere of 15 mm in radius centered at the peak of each cluster ($p<0.05$ FWE).

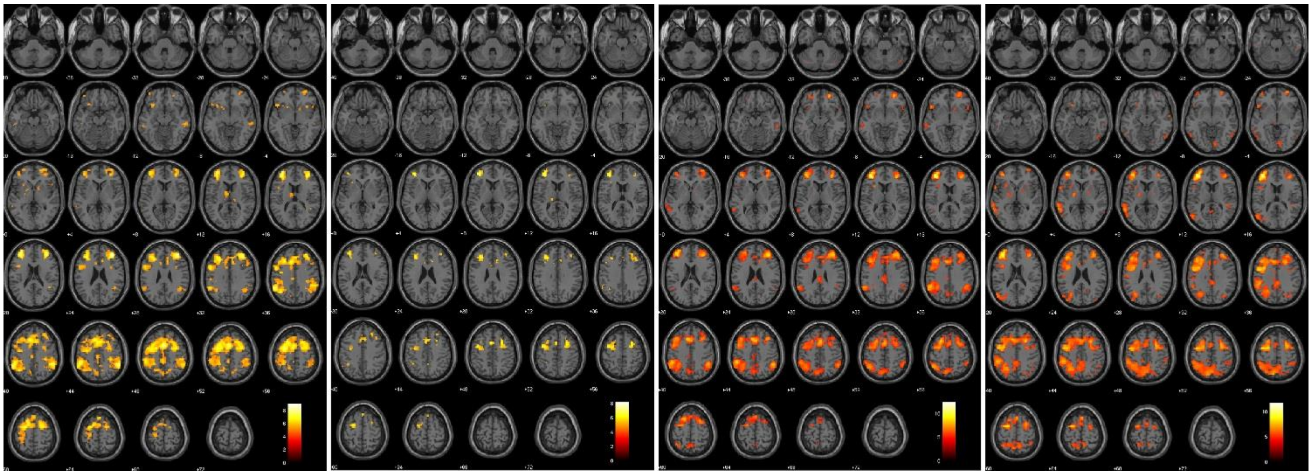

Figure S2 Axial slice views of PPI activity in a 2 group (low SA and high SA) x 2 condition (Positive>baseline and Neutral>baseline) ANOVA, thresholded at $p<0.05$, FWE corrected. Panels from left to right: Positive>baseline, low SA group; Neutral>baseline, low SA group; Positive>baseline, high SA group; Neutral>baseline, high SA group. PPI seed was a sphere of 8 mm radius centered at the left VLPFC (MNI: [-33, 41, 13]).

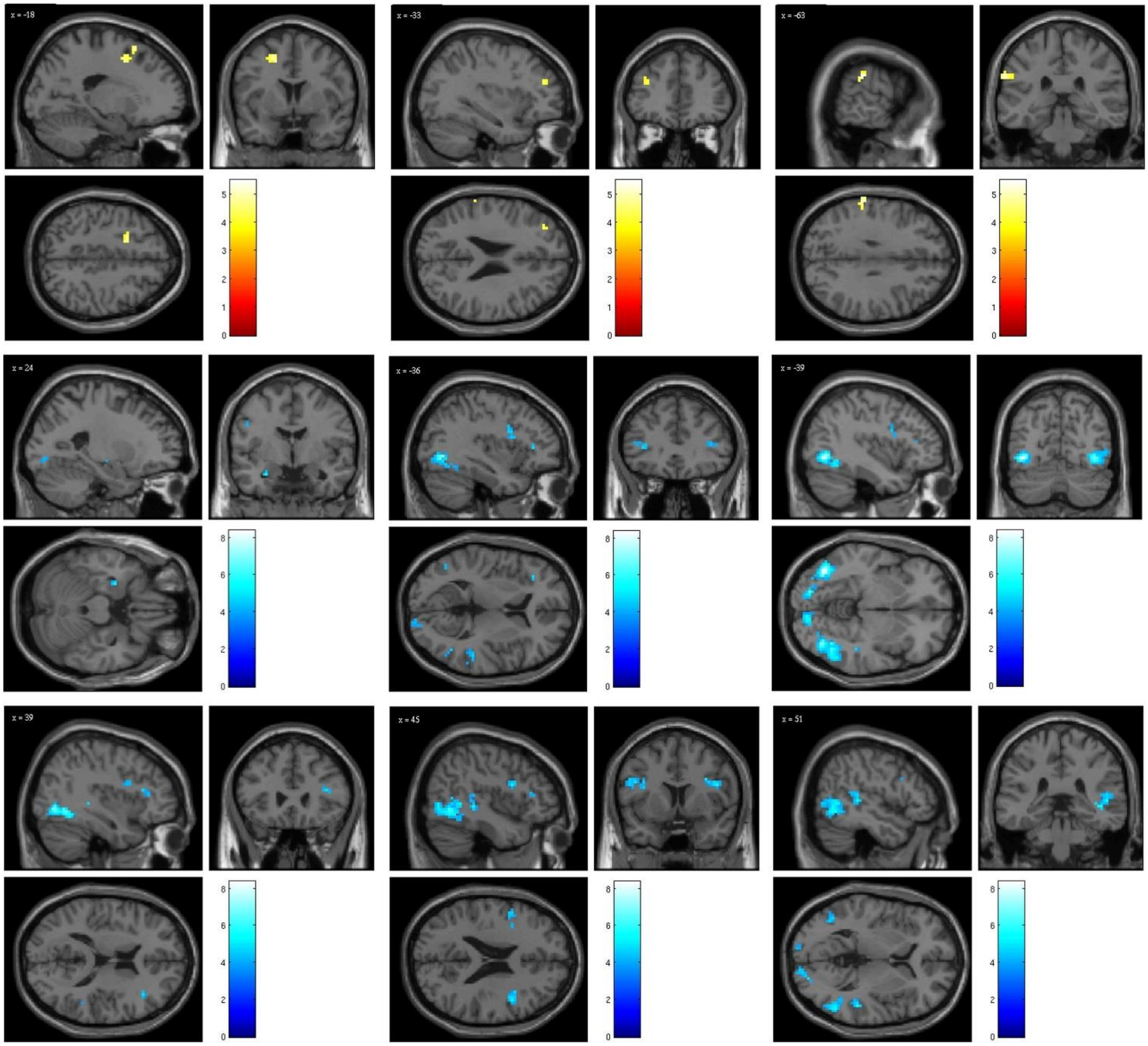

Figure S3 PPI activity when viewing positive social emotions versus neutral emotion in low and high SA groups. Shown are selected orthogonal views of one-sample T-test results shown in Table 2. Data were thresholded at $p<0.001$ uncorrected for multiple comparisons with cluster size threshold $k=216\ mm^3$.   PPI seed region was a sphere of 8 mm radius centered at the left VLPFC (MNI: [-33, 41, 13]). Shown in top row are active clusters with positive PPI activity (Positive>Neutral) in low SA group. Shown in bottom two rows are active clusters with negative PPI activity (Positive<Neutral) in high SA group.
